# The emerging Pannexin 1 signalome: a new nexus revealed?

**DOI:** 10.3389/fncel.2013.00287

**Published:** 2014-01-08

**Authors:** Leigh E. Wicki-Stordeur, Leigh A. Swayne

**Affiliations:** ^1^Division of Medical Sciences, University of VictoriaVictoria, BC, Canada; ^2^Department of Biology, University of VictoriaVictoria, BC, Canada; ^3^Department of Biochemistry and Microbiology, University of VictoriaVictoria, BC, Canada; ^4^Department of Cellular and Physiological Sciences and Island Medical Program, University of British ColumbiaVancouver, BC, Canada

**Keywords:** Pannexin 1, Panx1, pannexins, interactome, P2X7, inflammasome, cytoskeleton

## Abstract

Pannexins (Panxs) are a family of single-membrane, large-pore ion, and metabolite permeable channels. Of the three Panx proteins, Panx1 has been most extensively studied, and has recently emerged as an exciting, clinically relevant target in many physiological and pathophysiological settings. This channel is widely expressed across various cell and tissue types; however its links to precise signaling pathways are largely unknown. Here we review the current literature surrounding presently identified Panx1–protein interactions, a critical first step to unraveling the Panx1 signalome. First we elucidate the reported associations of Panx1 with other ion channels, receptors, and channel signaling complexes. Further, we highlight recently identified Panx1–cytoskeleton interactions. Finally, we discuss the implications of these protein–protein interactions for Panx1 function in various cell and tissue types, and identify key outstanding questions arising from this work.

## WHAT IS THE VALUE OF UNRAVELING THE PANX1 SIGNALOME?

Panx1 forms large pore channels permeable to ions and small molecules, and is a clinically relevant protein in inflammatory conditions (Kanneganti et al., 2007; Silverman et al., 2009; Gulbransen et al., 2012; Diezmos et al., 2013), stroke (Thompson et al., 2006; Bargiotas et al., 2011; Bargiotas et al., 2012; Dvoriantchikova et al., 2012), and cancer (Lai et al., 2007; Cowan et al., 2012; Penuela et al., 2012). Panx1 is ubiquitously found in many cell and tissue types throughout the body, while Panx2 and Panx3 exhibit slightly more restricted organ and tissue expression patterns. Because of its widespread distribution, future attempts to develop Panx1-based therapeutic strategies will optimally incorporate tissue/cell type specificity to minimize side effects. Knowledge of the signaling pathways in which Panx1 participates, more precisely, its unique cell- and tissue-specific protein interaction partners, will be important for the development of such targeted therapeutic strategies. Thus, not only will unraveling the web of the Panx1 signalome be key for understanding the depth and breadth of its tissue and cell-specific functions, it will also be critically important for Panx1-based drug development.

## PANX1 PERSPECTIVES, THEN AND NOW

Panx1 was cloned in 1998, and the pannexin family was first described as putative gap junction proteins in 2000 (Panchin et al., 2000) based on their homology to innexins, the invertebrate gap-junction forming proteins. Of the three Panx family members, Panx1 has been the primary focus of research. A large body of work has developed the current consensus that Panx1 forms single membrane channels (recently reviewed MacVicar and Thompson, 2009; Sosinsky et al., 2011). Individual four-pass transmembrane domain Panx1 subunits come together as hexamers. Panx1 channels appear to be relatively non-selective, permitting the passage of small ions and molecules up to one kilodalton in size. Panx1 is perhaps most well-known for its role in facilitating ATP release from a variety of cell types under both physiological and pathophysiological contexts, by several mechanisms of activation (recently reviewed Sandilos and Bayliss, 2012).

**FIGURE 1:**
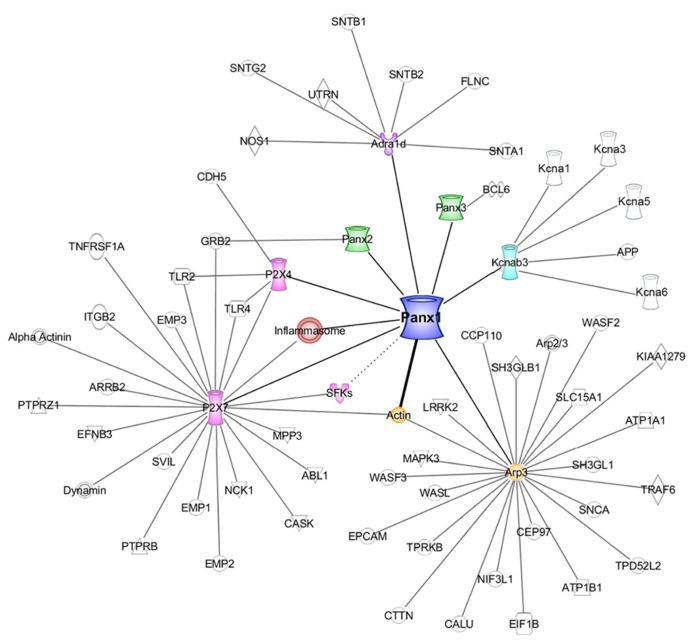
**The Panx1 interactome.** A diagram generated using Ingenuity Pathways Analysis [IPA; Ingenuity Systems (Qiagen), Redwood City, CA, USA] representing a curated list of the Panx1 interacting proteins, and the subsequent IPA-derived interaction networks of these interactors (excluding actin). The thick line between Panx1 and actin represents a direct interaction. The dotted line between Panx1 and SFKs indicates a likely link between the two proteins, however, no physical interaction data is yet available.

There are still significant gaps in the knowledge of the molecular mechanisms regulating Panx1 function and its downstream effects. Since its discovery, only a handful of studies have captured small snapshots of the Panx1 interactome. Here we unite these somewhat disparate pieces in order to develop a clearer picture of the cellular signaling network of Panx1. We also highlight data from our recent study that included the first unbiased proteomics analysis of Panx1 interacting proteins (Wicki-Stordeur and Swayne, 2013). In this minireview, we have grouped these interacting proteins into two major categories: (1) ion channels, receptors, and their signaling complexes and (2) cytoskeletal proteins (summarized in **Table 1; Figure 1**).

**Table 1:** Summary of Panx1 protein interaction partners, supporting evidence, functional significance, and relevant references.

Interactor	Evidence	Function	Reference
**Ion channels and receptors (and signaling complexes)**
Pannexin 2 (Panx2)	MYC IPs (OE)	Modification of Panx1 channel	Bruzzone et al. (2003, 2005)
	Functional coupling (OE)	function	Penuela et al. (2009)
	Panx1 and Panx2 IPs (OE)	Panx2 trafficking and localization	Ambrosi et al. (2010)
	Purified channels (OE)		
Pannexin 3 (Panx3)	Co-loc (OE and Endg)	Unknown	Penuela et al. (2009)
	Panx1 and Panx3 IPs (OE)		
Purinergic receptor, P2X, ligand-gated ion channel 7 (P2X7 receptor)	MYC IP (OE)	Panx1 forms the large pore of the P2X7 receptor complex known as the “death pore” Inflammasome activation	Pelegrin and Surprenant (2006), Locovei et al. (2007), Iglesias et al. (2008), Silverman et al. (2009), Poornima et al. (2012), Xu et al. (2012), Kanjanamekanant et al. (2013), Wang et al. (2013)
	Functional coupling (OE)		
	EGFP IP (OE)		
	Panx1 and P2X7 IPs		
	P2X7 IP		
	Panx1 IP		
	MYC and FLAG IPs (OE)		
Inflammasome (associated with P2X7)		Inflammasome activation	
NOD-like receptor family, pyrin-domain containing 1 (NLRP1)	Panx1 IP	(NLRP and/or cell-type dependent)	Silverman et al. (2009)
NOD-like receptor family, pyrin-domain containing 2 (NLRP2)	NLRP2 IP		Minkiewicz et al. (2013)
NOD-like receptor family, pyrin-domain containing 2 (NLRP3)	FLAG and HA IPs		Wang et al. (2013)
Apoptosis-associated speck-like protein containing CARD (ASC)	Panx1 and ASC IP ASC IP		Silverman et al. (2009), Minkiewicz et al. (2013)
X-linked inhibitor of apoptosis (XIAP)	Panx1 IP		Silverman et al. (2009)
Caspase 1 (Casp1)	Panx1 IP		Silverman et al. (2009)
Caspase 11 (Casp11)	Panx1 IP		Silverman et al. (2009)
Purinergic receptor, P2X, ligand-gated ion channel 4 (P2X4 receptor)	P2X4 IP	ATP-induced ROS production	Hung et al. (2013)
Voltage-gated potassium channel subunit beta-3 (Kcnab3)	B2H and co-loc HA and MYC IPs	Modulating Panx1 redox sensitivity and Panx1 inactivation	Bunse et al. (2005, 2009)
α-1D adrenergic receptor (Adra1d)	Panx1 and Adra1d IPs	Panx1 activation coupled to adrenoreceptor function	Billaud et al. (2011)
		Possible role in blood pressure regulation	
**Cytoskeletal Proteins**
Actin	Panx1 (OE) IP and BA	Panx1 trafficking and stabilization	Bhalla-Gehi et al. (2010), Wicki-Stordeur and Swayne (2013)
	Panx1 IP and co-loc (OE)		
Actin-related protein 3 (Arp3)	GFP IP and LC-MS/MS	Unknown	Wicki-Stordeur and Swayne (2013)
	Panx1 IP and co-loc (OE)		

*B2H, bacterial two-hybrid; BA, binding assay; Co-loc, Co-localization; Endg, endogenous; IP, immunoprecipitation; OE, overexpressed; LC-MS/MS, high performance liquid chromatography coupled to tandem mass spectrometry analysis.*

## ION CHANNELS, RECEPTORS, AND THEIR SIGNALING COMPLEXES

### PANX1–PANNEXIN INTERACTIONS

An early starting point was other members of the Pannexin protein family (Bruzzone et al., 2005). The initial impetus for investigation into Panx–Panx interactions likely arose from a rich literature detailing the intermixing of connexins (reviewed in Koval, 2006), the vertebrate gap junction protein family. Connexins share structural similarity to pannexins, but no sequence similarity. The first identified Panx1 binding protein was its family member, Panx2 (Bruzzone et al., 2005).

Panx2 is a much larger protein that Panx1, owing to its exceptionally longer C-terminus. It is particularly enriched in the brain (Baranova et al., 2004). The discovery of this interaction by Bruzzone et al. (2005) arose subsequent to an earlier observation by the same group, that depolarization-evoked currents in *Xenopus* oocytes injected with RNA for both Panx1 and Panx2 were significantly smaller and had modified gating kinetics in comparison with currents from *Xenopus* oocytes injected with Panx1 RNA alone (Bruzzone et al., 2003). The RNA encoding Panx2 itself did not lead to a depolarization-activated current. The authors proposed that Panx1/Panx2 heteromerization was the underlying cause for the difference in depolarization-evoked currents. Their later discovery of the interaction between epitope-tagged Panx1 and Panx2 supported this hypothesis (Bruzzone et al., 2005).

Key questions arose from these initial findings. For these findings to be physiologically relevant, Panx1 and Panx2 must be found in the same cells, and need to come into close proximity. Whether Panx1 and Panx2 are found in the same subcellular compartments is still an open question. To date, several studies have shown that ectopically and endogenously expressed Panx1 are found primarily at the cell surface, with some reports of intracellular expression. In contrast, ectopically (Lai et al., 2009; Penuela et al., 2009) and endogenously (Swayne et al., 2010; Wicki-Stordeur et al., 2013) expressed Panx2 are largely found primarily in intracellular compartments. Whether Panx2 forms functional channels at the cell membrane is contentious (but see Penuela et al., 2009). Further, if Panx2 forms functional channels, are they activated in the same way as Panx1 channels? Does the interaction occur early in the secretory pathway, and could it impact on the trafficking of Panx1?

Penuela et al. (2009) later demonstrated that co-expression with Panx1 in HEK293T cells dramatically increased the cell surface expression of Panx2, measured by cell surface biotinylation and confocal microscopy. In HEK 293T cells ectopically co-expressing Panx1 and Panx2, the authors confirmed reciprocal co-precipitation of the two proteins. Interestingly, immunoprecipitation of Panx2 co-precipitates the immature glycosylation species of Panx1 specific to the early secretory pathway (gly0 and gly1). This suggests the proteins are able to interact early in the secretory pathway, not long after translation. Somewhat paradoxically, the ability of Panx1 to increase the cell surface expression of Panx2 suggests that Panx1/Panx2 reaches the plasma membrane. In a similar set of experiments, this group also demonstrated overlapping distributions of Panx1 and Panx3 using confocal microscopy, and an interaction by immunoprecipitation. The extent of Panx1 and Panx3 co-precipitation was modulated by the glycosylation state of Panx1, but the physiological implications of these findings have not yet been fully elucidated.

Interestingly, Ambrosi et al. (2010) found that purified Panx1/Panx2 heteromeric channels from insect cells were unstable over time (present at 1 h, but not 24 h post-purification). Additional experiments led the authors to suggest these findings could have resulted from size differences between Panx1 and Panx2, as well as experimentally identified differences in oligomeric symmetry (their study suggested that Panx1 forms hexamers while Panx2 forms octamers).

How Panx1 and Panx2 might functionally interact in cells remains to be determined. In the context of the nervous system, the distribution of Panx2 appears to change from intracellular to cell surface during the course of hippocampal neurogenesis (Swayne et al., 2010), but whether Panx1 and Panx2 have overlapping distributions in mature neurons is currently unknown. Recent work investigating the role of Panxs in stroke recovery has demonstrated that knockout of both Panx1 and Panx2 improves histological and behavioral outcomes (Bargiotas et al., 2011; Kindo et al., 2011). Thus elucidation of the functional and physical relationships between members of the pannexin family will provide us with both fundamental and clinically relevant information. Although much progress has been made, there are still many unanswered questions relating to the crosstalk between Panx glycosylation and trafficking and the role of the Panx family interactions in this respect.

### P2X7 RECEPTORS

It is perhaps not surprising that the physiological and pathophysiological roles of pannexins and P2 purinergic receptors are connected given the former is perhaps best known as an ATP release channel, and the latter act as receptors for ATP and its metabolite, ADP. Pelegrin and Surprenant (2006) first reported a physical and functional interaction between overexpressed Panx1 and P2X7 receptors in 2006. The authors also demonstrated that P2X7 receptor activation in macrophage led to the opening of a large pore permeable to dye and IL-1β release. Based on their data, the authors concluded that Panx1 channels formed the large pore previously ascribed to P2X7 receptors alone. Subsequent work by Locovei et al. (2007) in the *Xenopus* oocyte heterologous expression model further supported this work. They showed that P2X7 receptors formed a non-selective ion channel, but co-expression with Panx1 was necessary to form a larger channel referred to as the “death pore.” Poornima et al. (2012) also reported an interaction between Panx1 and P2X7 receptors overexpressed in N2a cells. Recently, Kanjanamekanant et al. (2013) demonstrated an interaction between endogenous Panx1 and P2X7 receptor in human periodontal ligament cells using reciprocal immunoprecipitations, and found this interaction was strengthened following mechanical stress. Interestingly, Xu et al. (2012) observed a functional interaction only between Panx1 and the P2X7a receptor splice variant using dye uptake studies in overexpressing HEK293 cells. It now appears that such a Panx1-P2X7 receptor complex may also involve P2X4 receptor components in some cell types, as Hung et al. (2013) recently co-precipitated these three proteins from immortalized gingival epithelial cells.

Extending on the earlier findings by Pelegrin and colleagues, in the J774 monocyte macrophage cell line derived from a BALB/C mouse tumor, Iglesias et al. (2008) showed that Panx1 activation was tightly coupled to P2X7 receptor activation via a Panx1/P2X7 receptor complex involving a Src family tyrosine kinase (SFK). This group demonstrated interaction between the endogenously expressed proteins and were able to block the functional interaction with a membrane permanent TAT-P2X7 peptide targeting the SH3 domain of the P2X7 receptor. Interestingly, Weilinger et al. (2012) have further linked SFKs more directly to Panx1 function using a Panx1 C-terminal competitive peptide strategy; however a physical interaction between the two proteins has not yet been illustrated. Alberto et al. (2013) observed conflicting findings with respect to the Panx1-P2X7 receptor coupling; in primary mouse and rat peritoneal macrophage cultures, RNAi targeting Panx1, as well as probenecid and carbenoxolone treatment to block Panx1, had no effect on ATP-induced P2X7 currents. It should be noted that these two studies used substantially different recording solutions. More importantly, there are likely substantial differences arising from the use of two different macrophage models. Macrophages are found in all tissues. As a result of their extreme inherent plasticity, these distinct tissue-subsets have a high level of diversity in terms of gene expression and functional capabilities (Wynn et al., 2013). Even within peritoneal macrophages, it has been recently revealed that there are two physical, functionally and developmentally distinct subsets (Ghosn et al., 2010). Thus, the Panx1/P2X7 receptor relationship in macrophages is likely state- and subset-dependent. Moreover microglia, the macrophage-like resident immune cells of the brain, also possess a P2X7-Panx1 functional unit. Rigato et al. (2012) recently observed that microglial proliferation is dependent on P2X7 receptor and not Panx1, however, initial microglial activation and/or recruitment may still possess a Panx1 component.

#### The inflammasome, P2X7 receptors, and Panx1 in cytokine activation and immune cell recruitment

Interestingly, Panx1 has recently been linked to the inflammasome, a large cytoplasmic complex involved in cytokine activation and immune cell recruitment (Martinon et al., 2002, 2009). P2X7 receptors and Panx1 have been reported to be associated with inflammasomes in a variety of cell types (Silverman et al., 2009). As critical components of the inflammasome, Nod-like receptor proteins (NLRPs) link the detection of “danger signals” [or danger associated molecular pattern molecules (DAMP)], arising under scenarios such as metabolic stress, to proteolytic activation of the pro-inflammatory cytokines IL-1β and IL-18. Other key complex proteins include the adaptor protein ASC (Apoptosis-associated speck-like protein containing a CARD), and the inflammatory caspases-1 and -11. While P2X7 receptors are established inflammasome components, a recent report (Silverman et al., 2009) in cortical neurons revealed the potential involvement of Panx1. Silverman and colleagues immunoprecipitated Panx1 from cultured primary cortical neurons. Major components of the neuronal inflammasome including the P2X7 receptor, NLRP1, ASC, caspase-1, caspase-11, and X-linked inhibitor of apoptosis protein (XIAP) co-precipitated with Panx1. Reciprocally, ASC co-precipitated Panx1 as well as NLRP1, caspase-1, caspase-11, and XIAP. The authors further showed that elevating extracellular potassium above the normal resting range opened Panx1 channels leading to caspase-1 activation, and that this was sensitive to the Panx1 blocker probenecid. They determined that potassium-dependent activation of Panx1 was independent of changes in the membrane potential, which suggested that stimulation of inflammasome signaling was mediated by an allosteric effect of potassium binding to Panx1.

Minkiewicz et al. (2013) recently described a novel inflammasome in human astrocytes. They detected NLRP2 protein in human astrocytes and investigated the putative involvement of Panx1 in an NLRP2-based astrocytic inflammasome. Immunoprecipitation of NLRP2 from human primary astrocyte cultures co-precipitated Panx1, ASC, caspase-1, and P2X7 receptor. Reciprocally Panx1, P2X7 receptor, NLRP2, and caspase-1 co-precipitated with ASC. A more recent study (Wang et al., 2013) showed that while overexpressed Panx1 and NLRP3, or Panx1 and P2X7 receptors co-precipitated, overexpressed ASC and Panx1 did not interact in HEK293T cells. Interestingly, there were no deficiencies in NLRP3-inflammasome activation in macrophage of Panx1 knock-out mice (Qu et al., 2011; Wang et al., 2013; but see, Hung et al., 2013).

Gulbrandsen and colleagues confirmed *in vivo* Panx1 involvement in the NLRP3 inflammasome. They found that blocking Panx1 channels in enteric neurons ameliorates some of the effects of experimental colitis, including neuronal death (Gulbransen et al., 2012). Further, enteric neuronal death was dependent on P2X7 receptor, ASC, and caspase function, but not that of NLRP3, implying that a separate Panx1-inflammasome complex may be involved. Therefore, whether Panx1 is universally involved in inflammasome complexes may be characterized by cell type specific NLRP expression and utilization. Furthermore, the precise functional role of the physical association between Panx1 and inflammasome components remains to be determined.

### POTASSIUM CHANNEL AUXILIARY SUBUNIT Kvβ3

One of the initial Panx1 interactome studies was performed by Bunse et al. (2005). They employed an *E. coli* two-hybrid system to search for potential Panx1 interactors. This study identified the potassium channel accessory subunit, K_v_β3 (Kcnab3) as a putative interactor with the Panx1 C-terminus. K_v_β3 interacts with the shaker related voltage-gated potassium channels K_v_1.1, 1.3, 1.5, and 1.6 to modify voltage-dependent activation, and inactivation kinetics (Leicher et al., 1998; Bahring et al., 2004; Tipparaju et al., 2012). A follow-up study confirmed the Panx1-K_v_β3 interaction in a double-overexpression system by co-precipitation for the tagged proteins. An additional functional investigation indicated that ectopically expressed Panx1 currents became somewhat desensitized to redox- and pharmacological-based inhibition upon co-expression of K_v_β3 in oocytes (Bunse et al., 2009). The authors postulated that K_v_β3 is important for regulating Panx1 channel function by modulating its sensitivity to redox potentials. Bunse et al. (2011) further confirmed redox modulation of Panx1 activity, however, the molecular mechanisms underlying this sensitivity remain unknown. This will likely prove to be important in pathological contexts such as stroke and hypoxia, where redox signaling plays an important role (reviewed in Valko et al., 2007).

### α1D-ADRENORECEPTOR

Because of the established role of Panx1 in the release of ATP, a signaling molecule important for vasoconstriction, Billaud et al. (2011) performed Panx1 immunoprecipitations from thoracodorsal resistance arteries to examine its role in vascular smooth muscle cell communication. They identified an interaction between endogenous Panx1 and the α1D-adrenoreceptor. Further, they noted that ATP release via Panx1 was necessary to facilitate phenylephrine-evoked α1D-adrenoreceptor-mediated vessel constriction. Moreover, results from a HEK293 expression system indicated a Panx1-dependent component to ATP release evoked by α1D-adrenoreceptor activation (Sumi et al., 2010). Taken together, these studies indicate a mechanism for Panx1 activation downstream of adrenoreceptor stimulation, and imply a role for this channel in systemic blood pressure regulation.

## CYTOSKELETAL PROTEINS

Perhaps unsurprisingly, due to its known mechanosensitive nature, Panx1 has recently been found to associate with cytoskeletal proteins. Bhalla-Gehi et al. (2010) initially noted a role of the actin-based microfilament cytoskeleton in the cell-surface trafficking and stabilization of Panx1. Further study revealed an interaction between ectopically expressed Panx1 and actin. Interestingly, *in vitro* binding assays with purified proteins revealed the Panx1 C-terminus as the region responsible for the direct interaction.

More recently, our group confirmed this Panx1-actin association with endogenous Panx1 immunoprecipitations from a neuroblastoma cell line (Wicki-Stordeur and Swayne, 2013). Moreover, immunofluorescence and confocal microscopy illustrated co-localization of Panx1-EGFP with actin.

Our study also uncovered several novel Panx1 interactors. We performed immunoprecipitations from Panx1-EGFP expressing cells, coupled to mass spectrometry-based identification. From these, a putative association of Panx1 with actin-related protein 3 (Arp3) was revealed. This interaction was confirmed through endogenous Panx1 co-precipitations, and co-localization by immunofluorescence. Arp3 is a component of the large Arp2/3 actin-regulating complex involved in nucleation and branching of microfilaments (reviewed in Firat-Karalar and Welch, 2011). Because of its key role in controlling the dynamic actin cytoskeleton, the Arp2/3 complex is key in several cellular processes dependent on actin remodeling, such as filopodia (Korobova and Svitkina, 2008; Spillane et al., 2011) and lamellipodia (Ingerman et al., 2013) formation, cell migration (Sawa et al., 2003; Schaefer et al., 2008), and neurite outgrowth (Schaefer et al., 2008; Firat-Karalar et al., 2011).

Using gene ontology (GO) analysis we determined that 10% of the mass spectrometry-identified putative Panx1 interactors in neuroblastoma cells fell under a cytoskeleton classification. While most of these have yet to be validated, the large proportion of cytoskeletal interactors implies a significant role for the cytoskeleton in regulation of Panx1 trafficking and function. Potentially further connecting Panx1 with the cytoskeleton and other ion channels, a novel interaction between stomatin and Panx1 has also recently been identified that inhibits Panx1 currents (Zhan et al., 2012). Much work will clearly be needed to begin to untangle the complexities of emerging Panx1 nexus.

## CONCLUDING REMARKS

The Panx1 interactome is beginning to emerge, however, many key questions remain unanswered. For instance, which of the Panx1 interactions are direct, and which occur indirectly through other bridging proteins? At present, the Panx1-actin relationship stands alone as the only proven direct interaction. The functional significance of many of these interactions is also currently unknown. Moreover, questions arise regarding the tissue and cell-type specificity of these interactions. For example, the Panx1-inflammasome connection not only seems to depend on which NLRP is forming the complex, but also on the cell type in question. Finally, little is understood regarding alterations to the Panx1 interactome under pathophysiological conditions, such as stroke. In order to fill such knowledge gaps in our understanding of the role of Panx1 in both physiological and pathophysiological settings, the Panx1 interactome must be more fully elucidated and understood. In this way we will uncover key molecular players involved in Panx1 regulation and function, and thus be able to consider Panx1 as a viable therapeutic target within clinical settings.

## AUTHOR CONTRIBUTIONS

Leigh E. Wicki-Stordeur and Leigh A. Swayne wrote and revised the manuscript. Leigh E. Wicki-Stordeur created the Table and Figure. Both authors approve of the manuscript and its contents.

## Conflict of Interest Statement

The authors declare that the research was conducted in the absence of any commercial or financial relationships that could be construed as a potential conflict of interest.
